# Prophylactic and Therapeutic Efficacy of Avian Antibodies Against Influenza Virus H5N1 and H1N1 in Mice

**DOI:** 10.1371/journal.pone.0010152

**Published:** 2010-04-13

**Authors:** Huan H. Nguyen, Terrence M. Tumpey, Hae-Jung Park, Young-Ho Byun, Linh D. Tran, Van D. Nguyen, Paul E. Kilgore, Cecil Czerkinsky, Jacqueline M. Katz, Baik Lin Seong, Jae Min Song, Young Bong Kim, Hoa T. Do, Tung Nguyen, Cam V. Nguyen

**Affiliations:** 1 International Vaccine Institute, Seoul, Korea; 2 Influenza Division, National Center for Immunization and Respiratory Diseases, Centers for Disease Control and Prevention, Atlanta, Georgia, United States of America; 3 Department of Biotechnology, College of Engineering, Yonsei University, Seoul, Korea; 4 Department of Animal Biotechnology, College of Animal Bioscience and Technology, Konkuk University, Seoul, Korea; 5 National Centre for Veterinary Diagnostics, Department of Animal Health, Hanoi, Vietnam; 6 Department of Microbiology, University of Alabama at Birmingham, Birmingham, Alabama, United States of America; Institute of Molecular and Cell Biology, Singapore

## Abstract

**Background:**

Pandemic influenza poses a serious threat to global health and the world economy. While vaccines are currently under development, passive immunization could offer an alternative strategy to prevent and treat influenza virus infection. Attempts to develop monoclonal antibodies (mAbs) have been made. However, passive immunization based on mAbs may require a cocktail of mAbs with broader specificity in order to provide full protection since mAbs are generally specific for single epitopes. Chicken immunoglobulins (IgY) found in egg yolk have been used mainly for treatment of infectious diseases of the gastrointestinal tract. Because the recent epidemic of highly pathogenic avian influenza virus (HPAIV) strain H5N1 has resulted in serious economic losses to the poultry industry, many countries including Vietnam have introduced mass vaccination of poultry with H5N1 virus vaccines. We reasoned that IgY from consumable eggs available in supermarkets in Vietnam could provide protection against infections with HPAIV H5N1.

**Methods and Findings:**

We found that H5N1-specific IgY that are prepared from eggs available in supermarkets in Vietnam by a rapid and simple water dilution method cross-protect against infections with HPAIV H5N1 and related H5N2 strains in mice. When administered intranasally before or after lethal infection, the IgY prevent the infection or significantly reduce viral replication resulting in complete recovery from the disease, respectively. We further generated H1N1 virus-specific IgY by immunization of hens with inactivated H1N1 A/PR/8/34 as a model virus for the current pandemic H1N1/09 and found that such H1N1-specific IgY protect mice from lethal influenza virus infection.

**Conclusions:**

The findings suggest that readily available H5N1-specific IgY offer an enormous source of valuable biological material to combat a potential H5N1 pandemic. In addition, our study provides a proof-of-concept for the approach using virus-specific IgY as affordable, safe, and effective alternative for the control of influenza outbreaks, including the current H1N1 pandemic.

## Introduction

Highly pathogenic avian influenza A virus (HPAIV) of the H5N1 subtype that has emerged since 2004, resulted in more than 430 cases of laboratory-confirmed human infection in 15 countries with a death rate of more than 50% (www.who.int/csr/disease/avian_influenza/). H5N1 influenza virus remains a global threat because of its continued transmission among domestic poultry and wild birds. H5N1 influenza vaccines are now under development but none are yet available for human use [1]. The current H1N1 influenza pandemic was officially declared on June 11, 2009 by the World Health Organization (WHO) (http://www.who.int/csr/disease/swineflu) based on the rapid worldwide spread of the novel swine-origin pandemic influenza A (H1N1) 2009 virus (H1N1/09). As vaccine manufacturers scramble to produce new H1N1 vaccines for the upcoming influenza season, the limited global supply of the vaccine will require both prioritizing target groups for vaccination and exploring other interventions that can help reduce H1N1/09 virus transmission and disease severity, including the administration of antiviral agents (http://h1n1.nejm.org/). Passive immunization (the transfer of specific immunoglobulins/antibodies (Abs) to a previously non-immune recipient host) could offer an alternative strategy to prevent and treat influenza virus infection. Even after targeted vaccines become available, passive immunization could still have prophylactic effects and provides an additional countermeasure against influenza [2].

A number of attempts have been made to develop human monoclonal Abs (mAbs) against H5N1 influenza haemagglutinin (HA) using Epstein-Barr virus (EBV) immortalization of B cells isolated from patients infected with H5N1 [3], phage display [4], humanized mAbs [5], and human recombinant Abs [6]. Passive immunization based on mAbs, however, may require a cocktail of mAbs with broader specificity to provide full protection, since mAbs are generally specific for single epitopes. Polyclonal Abs that recognize multiple epitopes on the surface of microbes provide better protection and are less expensive compared to mAbs [7]. Chickens produce a unique immunoglobulin molecule called IgY that is functionally equivalent to mammalian IgG [8]. IgY are found in the sera of chickens and are passed from hens to the embryo via the egg yolk, imparting a high concentration of chicken IgY to the developing embryo [9]. Egg IgY have been used to prevent bacterial and viral infections [see review [10]] of the gastrointestinal tract and recently for protection against *Pseudomonas aeruginosa* infection of the respiratory tract of patients with cystic fibrosis (CF) [11]. However the effectiveness of IgY against influenza virus infection has not been explored.

The recent epidemic of HPAIV H5N1 virus has resulted in serious economic losses to the poultry industry, mostly in Southeast Asia (www.fao.org/docs/eims/upload/214194/rushton-comp.pdf). Therefore, many countries including China, Indonesia, Thailand, and Vietnam have introduced mass vaccination of poultry with H5N1 virus vaccines that controls the H5N1 epidemic to some extent [12]. Chickens immunized with recombinant H5 and/or inactivated H5N1 reassortant vaccines produced a high level of virus-specific serum Abs and were protected from H5N1 virus challenge [13]. Theoretically, H5N1-specific Abs are passed from hen to embryo and could be separated and used in humans for prevention against and therapy of H5N1 HPAIV infection and disease, respectively. Here, we tested the possibility that IgY isolated from consumable eggs available in supermarkets in Vietnam where mandatory H5N1 vaccination has been implemented, provide prophylaxis and therapy of HPAIV H5N1 infection and thus an alternative against potential A/H5N1 pandemic. Furthermore, we examined whether IgY isolated from eggs of hens immunized with inactivated H1N1 A/PR/8/34 (PR8) virus prevent influenza virus infection and cure the disease in mice. The results will provide a proof-of-concept for the approach using generated H1N1/09 virus-specific IgY to combat current H1N1 pandemic.

## Materials and Methods

### Animals

Female wild-type (WT) BALB/cAnNCrl (H-2^d^) mice were purchased at 6 to 8 weeks of age from Charles River Co. (Wilmington, MA) or the Jackson Laboratory (Bar Harbor, ME). All mice were maintained in specific pathogen–free barrier facilities. All experiments and animal procedures conformed to protocols approved by the Institutional Animal Care and Use Committees of Seoul National University, Yonsei University, Konkuk University, Seoul, Korea, and the United States Centers for Diseases Control and Prevention (US CDC), Atlanta, GA., USA. Hy-Line Leghorn hens purchased from Kyunggi Poultry Farm were housed in an animal facility at Konkuk University. All the hens were kept in rooms lightened for 16 h per day with constant temperature of 25°C.

### Cell lines

Madin-Darby canine kidney (MDCK) cells (ATCC, Manassas, VA) were maintained in standard complete Dulbecco's modified Eagle's medium (D-MEM) (Gibco, Grand Island, NY) containing 5% fetal bovine serum (FBS) and antibiotics.

### Viruses

Influenza virus strains, A/PR/8/34 (H1N1) (PR8) and A/Philippines/2/82/X-79 (H3N2) (A/Philippines) were prepared as previously reported [14]. Mouse-adapted viruses PR8 and A/Philippines, harvested from supernatants of mouse lung homogenates of intranasally infected mice were used for challenge. The H5N1 human influenza isolate A/Vietnam/1203/2004 (VN/1203) was obtained from the World Health Organization (WHO) influenza collaborating laboratory at the US CDC, Atlanta, GA. Inactivated reassortant avian H5N1 influenza virus (A/Goose/GD/96-derived, strain Re-1) (Harbin, China) was used for mass vaccination of poultry in Vietnam, and A/ck/Scotland/59 (H5N1) was used for determination of haemagglutination inhibition (HI) titers of sera and IgY from hens raised in Vietnam. The A/Aquatic bird/Korea/W81/2005 (H5N2), isolated from a wild bird in Korea in 2006, kindly provided by Dr. Young-Ki Choi, Chungbuk University, Korea, was adapted by multiple passages (15 times) in BALB/c mice. After final passage, a single plaque was isolated by three consecutive plaque purifications on MDCK cells, amplified in embryonated chicken eggs, and the LD_50_ of the H5N2 virus was determined in mice for challenge experiment. Avian H5N1 viruses were propagated in the allantoic cavity of 10-day-old embryonated hen's eggs at 37°C for 24 h to 30 h. The H5N1 human influenza isolate was incubated for an additional 10 h to 18 h. Allantoic fluid was pooled from multiple eggs, clarified by centrifugation, and frozen at −70°C until use. All experiments with HPAI (VN1203) virus were conducted under Biosafety Level 3 containment, including enhancements (BSL3+) required by the U.S. Department of Agriculture and the Select Agent [15].

### Eggs

Eggs laid by hens raised in the poultry unit of Konkuk University, Seoul, Korea and purchased from randomly selected supermarkets in Hanoi, Vietnam and Seoul, Korea and farms in Vietnam were used in experiments.

### Hen immunization

Twenty-five-week-old domestic Leghorn hens were immunized intramuscularly with heat-inactivated A/PR/8/34 (H1N1) mixed with Freund's adjuvant (FA) (Sigma, MO, USA). 5 µg of antigen was suspended in 250 µl of phosphate-buffered saline (PBS) and emulsified with an equal volume of complete FA. Incomplete FA was used for boosting immunizations. The hens were immunized three times with two weeks between the immunizations. The hen sera were collected eight weeks after the initial immunization, and eggs laid after last immunization were collected continuously. In some cases, immunized hens were boosted within a 3–4 months' interval to keep in hyperimmunized condition for a longer time period.

### Preparation of IgY

A rapid and simple water dilution method for extraction of IgY from egg yolk was adapted from the work by Akita and Nakai [16]. Briefly, the yolk from ten eggs (total volume, 120 ml) was separated from the white by egg separators and washed with deionized water. Each yolk sac was disrupted by inserting a needle and the yolk was allowed to drip through a nylon mesh into a measuring cylinder. The egg yolk was diluted 10 times with cold 3 mM HCl to give the suspension a final pH of 5 (adjusted with 10% acetic acid). The suspension was incubated for at least 6 h at 4°C before the supernatant containing the IgY was collected by centrifugation (10 000×*g* for 15 min at 4°C). Solid ammonium sulfate was added to reach 60% saturation (390 g/l) and the mixture was stirred in the cold for 15 min. Precipitate was collected by centrifugation and washed once with 60% saturated ammonium sulfate (SAS). The protein precipitate was dissolved in PBS and dialyzed three times against at least 10 volumes of PBS. Dialyzed IgY was adjusted to the original egg yolk volume (ten yolks equal 120 ml) pasteurized at 60°C for 30 minutes and stored at 4°C. The purity of the IgY preparations was determined by sodium dodecyl sulfate-polyacrylamid gel electrophoresis (SDS-PAGE) followed by Coomassie blue staining and is consistent with that obtained by others [10], [17], [18].

### Infection and treatment of mice

Fifty percent lethal dose (LD_50_) titers were determined by inoculating groups of eight mice intranasally with serial 10-fold dilutions of virus as previously described [19]. For infection, ketamine-anesthetized mice were inoculated intranasally with a lethal dose with 250 pfu (5× LD_50_) of A/PR/8/34 (H1N1) virus, 1,000 pfu (5× LD_50_) of A/Philippines (H3N2), 10× LD_50_ of VN/1203 (H5N1) or 5× LD_50_ A/Aquatic bird/Korea/W81/2005 (H5N2) resuspended in 50 µl PBS per animal. Ketamine-anesthetized mice were treated intranasally with 50 µl of IgY before or after infection. Death was defined when animals presented with more than a 25% weight loss, which required euthanasia and used as the endpoint in these studies.

### Virus titration

The 50% egg infectious dose (EID_50_) was determined by serial titration of virus stock in eggs, and EID_50_/ml values were calculated according to the method of Reed and Muench [20]. Human virus stocks were grown in MDCK cells as described previously [21], with viral titers determined by standard plaque assay. Determination of 50% tissue culture infectious dose (TCID_50_) was performed on MDCK cells by detection of viral protein according to Rowe et al. [22]. Briefly, 100 µl of freshly trypsinized MDCK cells (2×10^5^/ml) were incubated in Dulbecco's modified Eagle's medium containing 5% FBS and 1× of antibiotic-antimycotic solution (GIBCO) (complete DMEM) in 96-well Nunclon™ Surface plates (NUNC, Inc., Roskilde, Denmark) for 3 h at 37°C with 5% CO_2_. Cells were washed with serum free medium before adding serial dilutions of virus-containing samples in 100 µl of DMEM containing 2.5 µg/ml of trypsin. Plates were incubated for 18 h at 37°C with 5% CO_2_ before fixation of the cells with cold 80% acetone for 10 min. Plates were then washed with PBS/Tween 20 before addition of anti-NP IgG antibody (US CDC, Atlanta, GA) diluted 1/4,000 in PBS containing 1% bovine serum albumin and incubated at room temperature for 1 h. Goat anti-mouse IgG horseradish peroxidase-conjugated antibody (Southern Biotechnologies Associates, Inc., Birmingham, Ala.) was added for 1 h at room temperature. Reaction was developed by adding 100 µl of freshly prepared TMB (3,3′,5,5′-tetramethylbenzidine) substrate (BD Biosciences, Franklin Lakes, NJ) to each well, and the plates were incubated at room temperature for approximately 5 min. The reaction was stopped with 50 µl of 1 M sulfuric acid. The absorbance was measured at 450 nm (*A*_450_) with SPECTRAmax photometer (Molecular Devices, Palo Alto, CA). Wells having an absorbance reading greater than 3 standard deviations above the mean absorbance of wells containing only MDCK cells were scored positive for virus growth. The TCID_50_ of each stock virus was calculated by the method of Reed and Muench [20].

### Microneutralization (MN) assay

Neutralizing antibody titers were determined by microneutralization (MN) assays performed on MDCK cells following the procedure as previously described [22]. Briefly, 2-fold serially diluted samples were incubated with 100 TCID_50_ of viruses in 96-well cell culture plates at 37°C for 1 h before adding to MDCK cells. The presence of viral protein was detected by ELISA with anti-NP IgG antibody as described above. The neutralizing antibody titers were expressed as the reciprocal of the highest dilution of serum that gave 50% neutralization of 100 TCID_50_ of virus in MDCK cells. Positive serum control and negative cell controls with no serum were included on each plate.

### Hemagglutination-inhibition (HI) assay

Samples were treated with receptor destroying enzyme II (RDE, Denka Seiken Co., Ltd., Tokyo, Japan) at a final dilution of 1∶3 before being tested in HI assay. Two-fold serially diluted samples were incubated with equal volume containing 100 TCID_50_ of viruses in U-bottom 96-well microtiter plates at 37°C for 1 h. At the end of incubation, freshly prepared 1% chicken red blood cells (CRBC) were added, and plates were mixed by agitation, covered, and allowed to set for 1 h at room temperature. The HI titers were determined by the reciprocal of the last dilution which contained non-agglutinated CRBC. Positive and negative control samples were included on each plate.

### ELISA

The standard ELISA was performed for detection of anti-IgY in the sera of IgY-immunized mice. 96-well MaxiSorp^TM^ Nunc-Immuno plates (Nalgene Nunc International, Naperville, IL) were coated overnight with purified IgY (Gallus Immunotech, Ontario, Canada) at a concentration of 10 µg/ml. Dilutions of serum were incubated 2 h on coated and blocked ELISA plates. Bound immunoglobulins were detected with goat anti-mouse Ig (H+L) horseradish peroxidase-conjugated antibody (Southern Biotechnologies Associates, Inc., Birmingham, Ala.) At the end of the incubation (2 h at 37°C), TMB substrate was added and the reaction was stopped with an equal volume of 1 M sulfuric acid. The color developed was measured in a SPECTRAmax photometer at 450 nm. The reproducibility of the assay was ascertained by applying a control hyperimmune mouse serum on each plate. Assay results were expressed as end-point titration values which are determined by the last dilutions that are above cutoff for assay (OD 450 nm reaches plateau).

### Statistics

The data are expressed as the mean ± one standard error of the mean (SEM) and compared using a two-tailed student's *t-*test or an unpaired Mann Whitney U test available in Microsoft Excel software (Redmond, WA).

## Results

### HI and VN activities of IgY isolated from consumable eggs available in markets in Vietnam

We first tested the possibility that yolks from commercially available eggs in Vietnam, where mass vaccination of poultry against avian influenza H5N1 is mandatory, contain H5N1-speficific IgY. We determined H5-specific HI titers in the sera and yolks of the eggs obtained from a farm in Vietnam that was participating in a national mass vaccination program. IgY preparation was restored in PBS to the original volume of yolk. Indeed, H5-specific HI titers determined in yolks were comparable to those seen in sera of vaccinated hens (Table 1). Next, we determined the H5-specific HI titers of IgY isolated from eggs purchased in randomly selected supermarkets in Hanoi, Vietnam that offer safe foods with recorded origin. Consistently, 90% of eggs purchased in supermarkets contain H5-specific IgY at the levels comparable with those observed in sera of hens selected randomly from the farm that underwent supervised H5N1 vaccination (data not shown). IgY pooled from 10 eggs have comparable HI and VN titers (Table 1). In contrast, IgY separated from eggs laid by unimmunized hens or purchased in Korean markets where poultry are not vaccinated against avian influenza H5N1 contained no detectable H5-specific HI or VN activity (Table 1).

**Table 1:** Haemagglutination inhibition (HI) and virus neutralization (VN) titers in hen sera and egg yolks.

	Sera		IgY	
Immunization	HI (log_2_)	VN	HI (log_2_)	VN
Heat inactivated PR8	6*	320*	8*	320*
Inactivated A/Goose/GD/96. Sera and eggs collected from farm – set 1	5.3±1.5▴	ND	7	ND
Inactivated A/Goose/GD/96. Sera and eggs collected from farm – set 2	5.5±1.0▴	ND	7	ND
Consumable eggs from Vietnam supermarket - batch 1	NA	NA	7**/***	320**
Consumable eggs from Vietnam supermarket - batch 2 (vn045)	NA	NA	7***	ND
Unimmunized – consumable eggs from Korea	<2	ND	<2	<10

ND: Not done.

NA: Not available.

* A/PR/8/34 (PR8).

** A/Vietnam/1203/2004 (VN/1203 – H1N1).

*** A/Aquatic bird/Korea/W81/2005 (H5N2).

▴ Mean and SEM of log_2_ titers.

Two hens were immunized with heat inactivated PR8 (as described in Materials and Methods) and IgY was pooled from 15 collected eggs. Sera of hens raised in Vietnam farms were tested individually (10 samples in each batch) and IgY was pooled from 10 eggs in each batch. If not otherwise indicated A/ck/Scotland/59 (H5N1) was used for H5 virus-specific HI titration. Sera from two unimmunized hens and IgY pooled from 10 eggs obtained in Korea were used as negative control. Titers of VN antibody were determined as the reciprocal of the highest dilution of specimens that neutralized 100 pfu or TCID_50_ of virus in MDCK cell cultures.

### IgY derived from consumable chicken eggs in Vietnam provide protection against H5 influenza viruses

**Figure 1:**
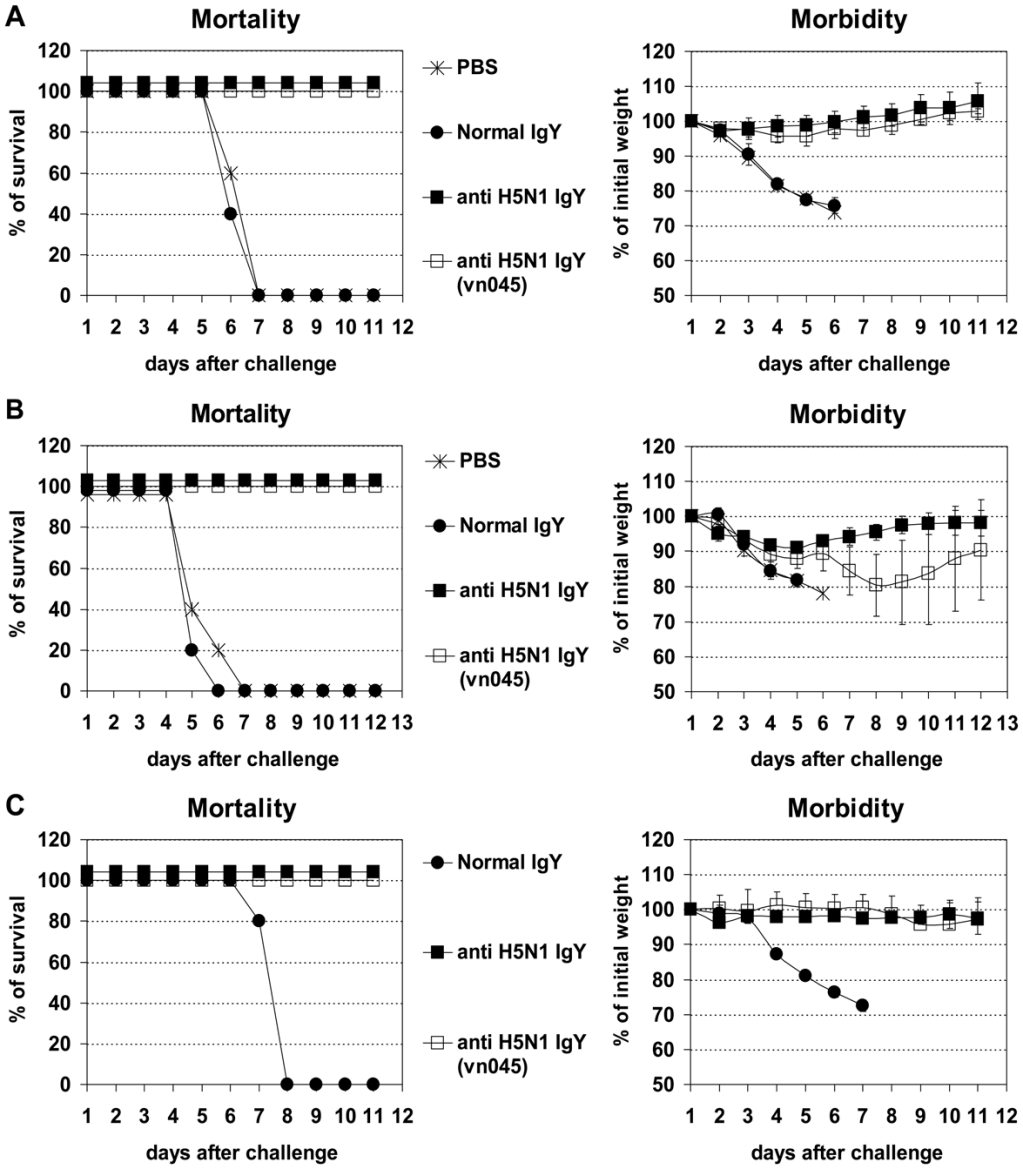
Protection against challenge with A/Aquatic bird/Korea/W81/2005 (H5N2). BALB/c mice were treated with H5N1-specific IgY [anti H5N1 IgY or different batch of anti H5N1 IgY (vn045)] at 6 hours before and 18, 42, and 66 hours after infection with H5N2 virus (Pre- and post-infection treatments, Fig. 1A); at 6, 30, 54, and 78 hours after infection (Post-infection treatments, Fig. 1B); or once at 6 hours before infection (Single pre-infection treatment, Fig. 1C. Five LD_50_ of mouse-adapted A/Aquatic bird/Korea/W81/2005 (H5N2) virus and 50 µl of IgY were used for intranasal infection and treatment, respectively. The values are the mean of 5–10 mice in each group.

We first used a mouse-adapted, low pathogenic avian influenza A virus (LPAIV) strain A/Aquatic bird/Korea/W81/2005 (H5N2), which shares 94.4% nucleotide sequence homology with HA (H5) but has different NA (N2) from the one used for mass immunization in Vietnam (reassortant avian H5N1 influenza virus A/Goose/GD/96-derived, strain Re-1) for challenge experiments in BALB/c mice. As shown in Fig. 1, complete protection against infection with avian H5N2 was achieved by intranasal administration with H5N1-specific IgY before or after the lethal infection (Fig. 1a and 1b). A single treatment with H5N1-specific IgY before inoculation was sufficient to protect animals completely from disease (Fig. 1c). Treatments with H5N1-specific IgY before and/or after infection with H1N1 PR8 that shares same type of NA but different HA did not prevent or cure the disease (data not shown).

**Figure 2:**
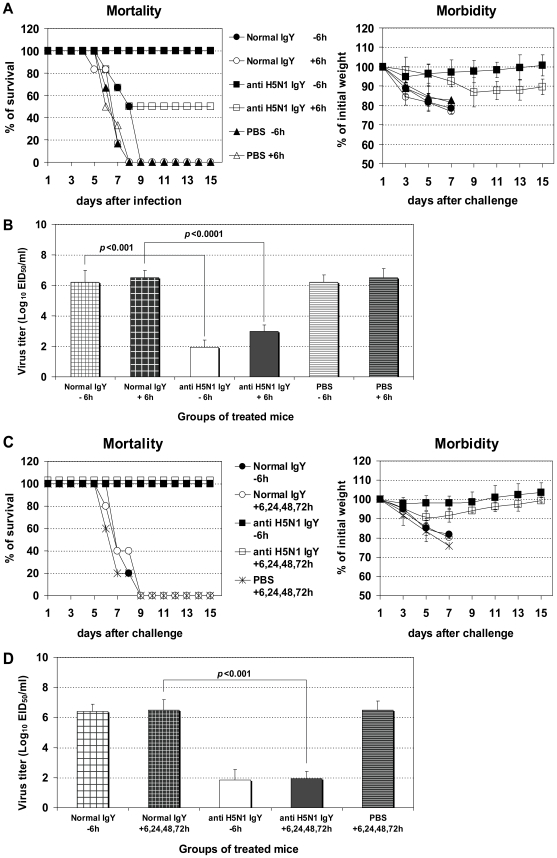
Protection against challenge with HPAIV H5N1. Morbidity and mortality of BALB/c mice that were treated with H5N1-specific IgY [anti H5N1 IgY] at 6 hours pre- (−6 h) or post- (+6 h) infection with VN/1203 (H5N1) virus (Single pre- or post-infection treatment, Fig. 2A - 6 mice per group); or at 6, 24, 48, and 72 hours after infection (Multiple post-infection treatments, Fig. 2C – 5 mice per group). Ten LD_50_ of VN/1203 H5N1 virus and 50 µl of H5N1-specific IgY were used for intranasal infection and treatment, respectively. The mice protected from disease did not die even after 3 weeks monitoring. Virus titers (EID_50_) in the lungs were determined on day 3 after infection (Fig. 2B and 2D). The values are the mean of 4 mice in each group. The group of mice receiving single pre-infection treatment was included as control.

Based on these results, we further examined whether protection against infection with HPAIV H5N1 strain, A/Vietnam/1203/2004, which was isolated from a fatal case, could be achieved. Animals treated intranasally with H5N1-specific IgY before infection displayed mild weight loss and recovered completely by the end of the first week after inoculation (Fig. 2a). Of note, animals treated with H5N1-specific IgY after H5N1 inoculation exhibited minimal weight loss during the first week after inoculation, and virus titers in the lungs were substantially reduced at day 3 after infection (Fig. 2b), but 50% of treated mice succumbed to infection during the second week after inoculation. It is possible that not all of the HPAIV H5N1 viruses were neutralized upon single treatment with IgY, and escaping viruses could have spread systemically to organs outside of the lungs. These viruses may reappear in lung tissue later when specific IgY are absent. Indeed, VN/1203 virus injected intravenously or into the brain can spread to the lungs [23]. To circumvent the virus escape, we administered multiple post-infection treatments with H5N1-specific IgY. As a result, all of the infected mice recovered completely by the second week post-infection (Fig. 2c), and virus titers in the lungs were substantially reduced to the level as seen in protected mice that received a single pre-infection treatment (Fig. 2d).

### H1N1 virus-specific IgY derived from immunized hens provide protection against lethal infection

**Figure 3:**
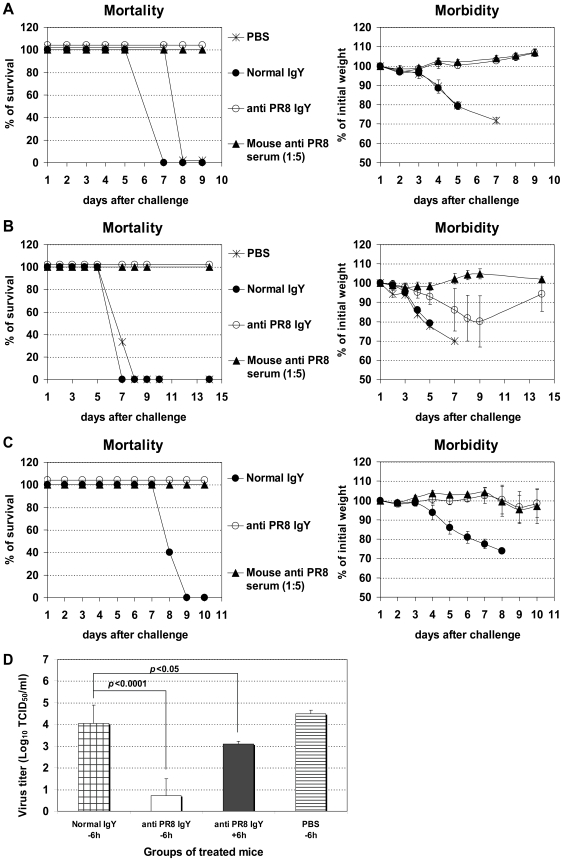
Protection against challenge with H1N1 PR8. BALB/c mice were treated with PR8-specific IgY (anti PR8 IgY) - as described in Materials & Methods at 8 hours pre- and 16, 40, and 64 hours post-infection (Pre- and post-infection treatments, Fig. 3A); at 8, 32, 56, and 80 hours post-infection (post-infection treatments, Fig. 3B); or once at 6 hours before infection (Single pre-infection treatment, Fig. 3C. Five LD_50_ of mouse-adapted PR8 and 50 µl of IgY were used for intranasal infection and treatment, respectively. Morbidity (body weight loss) and mortality were monitored daily until recovered animals regained their initial weight. The values are the mean of 5–10 mice in each group. Mortality is expressed as % of mice that survived the lethal infection. Virus titers in the lungs (TCID_50_) determined at day 3 after infection in mice treated with PR8 specific IgY at 6 hours before (−6 hrs) or after (+6 hrs) infection (Fig. 3D). The values are the mean of 8 mice in each group derived from 2 independent experiments. As control, a group of mice treated with mouse anti-PR8 serum (HI titer 1∶128) was included. All mice received the same amount of the IgY preparation of identical HI titer.

We further examined the protective effect of IgY prepared from eggs laid by hens immunized in the laboratory with heat-inactivated human influenza A H1N1 PR8 virus. PR8 virus is a common laboratory mouse-adapted influenza strain that serves as a model virus for current pandemic H1N1/09 virus and can be handled safely under Biosafety Level 2 (BSL2) conditions. We found substantial levels of haemagglutination inhibition (HI) and virus neutralization (VN) Abs in the sera and yolks derived from immunized hens (Table 1). When naïve mice were administered anti-PR8 IgY intranasally at 6–8 h before and 16, 40 and 62 h after infection (Fig. 3a) or only after infection with a lethal dose of PR8 virus (Fig. 3b), they were protected from disease or death, respectively. Mice receiving anti-PR8 IgY only after infection started to lose weight after the last IgY treatment (day 3 after infection) with the maximum weight loss occurring 9 days post-infection, however they recovered completely 14 days after the infection. Importantly, a single treatment at 6 h before lethal challenge prevented weight loss, a measure of morbidity, which was comparable with that seen in the control group receiving murine immune serum specific for PR8 virus (anti-PR8 serum) (Fig. 3c). The virus titers in the lungs of PR8-specific IgY-treated mice at day 3 after infection were significantly lower than those seen in untreated mice or in mice receiving normal IgY (Fig. 3d). The protection correlated with VN activity of the virus-specific IgY and virus clearance in the lungs of infected mice (Table 1 and Fig. 3c) suggesting that VN is the major mechanism of protection.

### IgY treatment induced anti-IgY Ab responses which do not prevent protection mediated by virus-specific IgY

**Figure 4:**
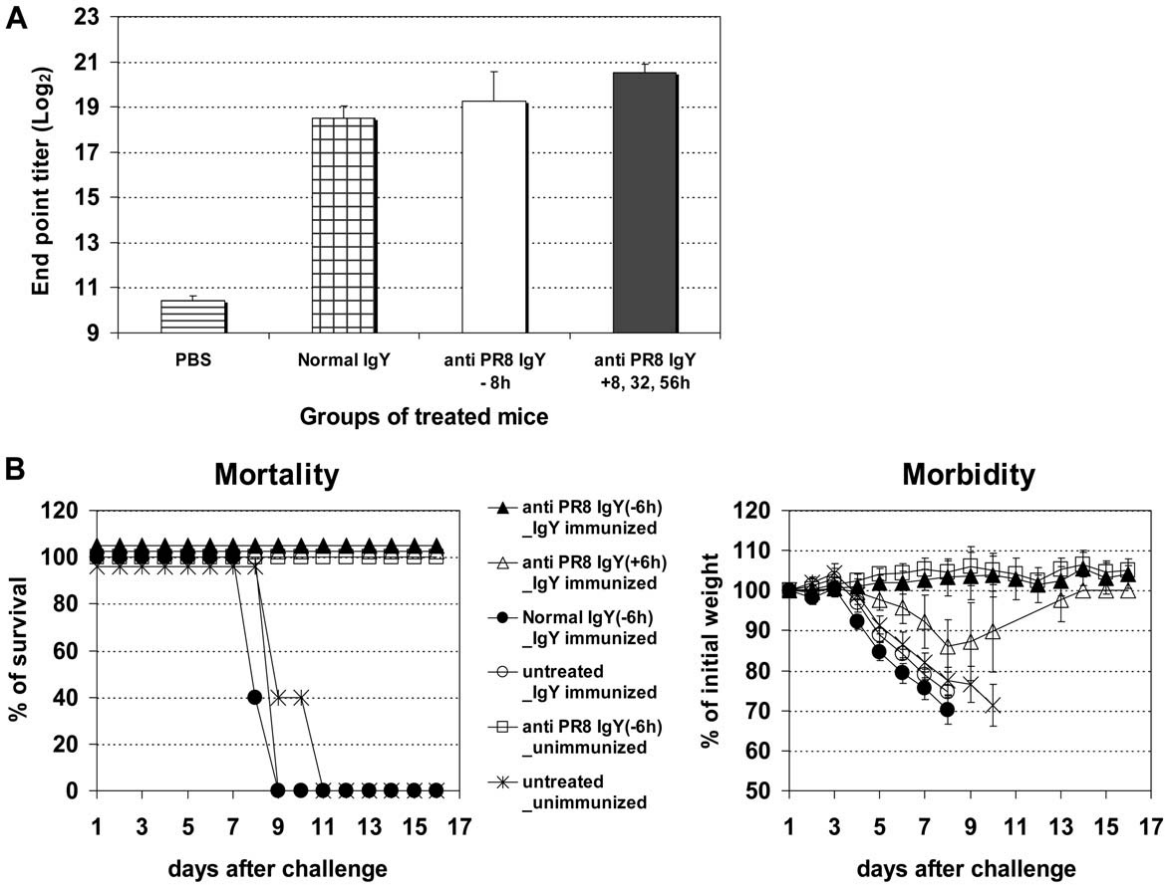
Induction of anti IgY Abs and IgY treatment in mice with pre-existing anti IgY. Anti IgY in the sera of mice immunized with normal IgY (IgY immunized), treated once intranasally with PR8-specific IgY 8 hours before (anti PR8 IgY −8 h) or three times after infection (anti PR8 IgY +8, 32, 56 h) (Fig. 4A). Endpoint titers (log_2_) were determined by ELISA. Morbidity and mortality of IgY-immunized mice treated with PR8-specific IgY (anti PR8 IgY) before (−6 hr) or after (+6 hr) infection with mouse-adapted PR8 (Fig. 4B). The values are the mean of 5–10 mice in each group.

Although raw eggs are consumed widely in many countries and uncooked egg components are used in the preparation of many foods, reports on the presence of anti-IgY Abs in humans have been limited to only two studies. One study demonstrated the presence of anti-IgY Abs in sera obtained from normal individuals [24], and the other study showed the absence of anti-IgY Abs in humans upon oral ingestion of IgY or consumption of raw egg components [25]. In mice, intravenous injection of IgY elicits a typical anti-IgY antibody response [26]. It is, however, not clear if administration of IgY in the respiratory tract induces an anti-IgY response. We examined sera obtained from IgY-treated mice for the presence of anti-IgY Abs. Indeed, detectable anti-IgY Abs were observed in animals that received IgY by single intranasal administration (Fig. 4a). There was no significant difference in the levels of anti-IgY Abs in mice receiving multiple or single administration of IgY.

**Figure 5:**
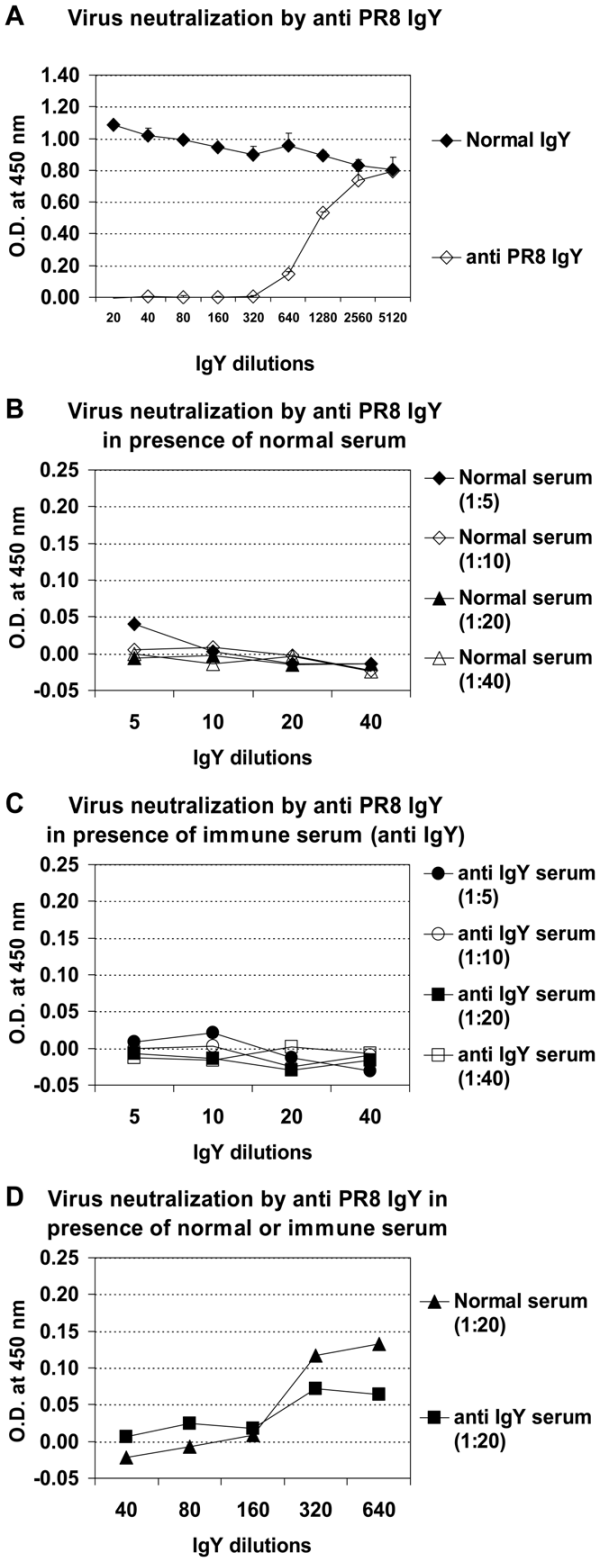
Anti-IgY Abs do not block neutralizing activity of virus specific IgY. PR8 virus neutralizing activity of PR8 specific IgY (anti-PR8 IgY) in the absence of anti-IgY serum was determined by microneutralization assay. VN titer of anti-PR8 IgY is 1∶320 at which the viral nuclear protein (NP) was not detected (Fig. 5A). In the presence of anti-IgY serum VN by anti-PR8 IgY was not abrogated by incubation with normal serum (Fig. 5B and 5D) or with anti-IgY serum (Figs. 5C and 5D). VN titer (1∶320) of anti-PR8 IgY was used in the assay. The optical density (O.D.) was determined at 450 nm (*A*_450_).

We next asked whether pre-existing anti-IgY Abs prevent virus-specific IgY-mediated protection. We immunized mice with normal IgY or immune IgY specific for particular subtypes. Three weeks later, serum Abs specific for IgY were determined by ELISA. One hundred percent of the immunized mice generated anti-IgY Abs at the level comparable to that of IgY-treated mice (Fig. 4a). Such IgY-immunized mice were then treated with virus-specific IgY before or after infection with a lethal dose of influenza virus. The results were almost identical to those obtained from treated, non-immunized mice (Fig. 4b), indicating that pre-existing anti-IgY Abs do not interfere with protection mediated by virus-specific IgY. We speculated that if IgY epitopes that bind anti-IgY Abs are not located in the virus-binding sites of the IgY, then anti-IgY Abs would not prevent the binding and/or neutralizing activities of virus-specific IgY. To investigate this question, we incubated murine anti-IgY serum with virus-specific IgY before adding to the HI and VN assays. Indeed, incubation with anti-IgY serum did not interfere with HI activity of the virus-specific IgY (not shown), indicating that anti-IgY Abs do not block virus binding by virus-specific IgY. Similarly, incubation with anti-IgY does not interfere with VN activity of the specific IgY (Fig. 5).

## Discussion

Several animal studies and a number of human studies of IgY against different pathogens have demonstrated preventive and prophylactic effects of IgY. Rotavirus-specific IgY provided protection against infection with bovine rotavirus both in calves and in mice, whereas anti-*Escherichia coli* IgY reduced mortality in newborn piglets [27]. Salmonellosis has been prevented by IgY both in neonatal calves and in a mice model. In humans, IgY against *Streptococcus mutans* decreased caries when used as a mouth rinse [28] and anti-*Helicobacter pylori* IgY reduced *Helicobacter* infections [29], [30]. IgY have been used for first time in humans to treat infection of respiratory tract, e.g. with *Pseudomonas aeuruginosa* [11], [31]. Our results indicate that when delivered intranasally IgY derived from hens immunized with inactivated influenza virus provide protection against lethal infection by neutralization of the viruses in the lungs. Importantly, we found that readily available IgY from consumable eggs in supermarkets in Vietnam provide prophylaxis and therapy of HPAIV H5N1 infection and thus offer an enormous source of valuable biological material to combat potential H5N1 pandemic. Initially, we used oral and intraperitoneal routes for treatments; however, no protective effect was observed by either route although IgY were detectable in the sera of IgY-treated mice after either route of delivery (data not shown). In humans, gargling could be an alternative to intranasal or pulmonary (aerosol) delivery since treatment with bacteria-specific IgY by gargling significantly reduced lung infections with *Pseudomonas aeuruginosa* in cystic fibrosis (CF) patients [11]. Current FDA approved intranasal delivery (spray) of licensed live attenuated influenza vaccine FluMist® (http://www.fda.gov/BiologicsBloodVaccines/Vaccines/ApprovedProducts/ucm094047.htm) that contains egg components could be a proof for safe intranasal delivery of IgY.

The efficacy of IgY seems to be specific for infection with viruses of the same HA type, since H5N1-specific IgY provided protection against infections with HPAIV H5N1 and H5N2 strains but failed to protect against infection with H1N1 PR8 virus that shares the same type of NA but not HA.

Although IgY treatment induced significant Ab response towards IgY, the anti-IgY, however did not interfere with the protective effectiveness of virus-specific IgY. The findings suggest that IgY treatment could be applied to persons who have developed anti-IgY Abs and that such a treatment strategy could be repeated if multiple treatment is required and necessary to protect infections against other pathogens later on. Indeed, the current protocol for treatment of *P. aeuruginosa* infection in the lungs of CF patients requires long-term repeated applications of anti-bacterial IgY [11]. Thus, our results provide a proof-of-concept that virus-specific IgY prevent influenza virus infection and cure the disease. The approach could be applied to generation of H1N1/09 -specific IgY to combat the current H1N1 pandemic. Since production of virus-specific IgY is relatively fast and cost-effective, IgY-based prophylaxis and therapy are practical for control of outbreaks with newly emerging influenza viruses.

The approach of using specific IgY for prevention and therapy of influenza virus infection offers an alternative to current immunotherapy, which uses HPAIV H5N1 convalescent plasma [32], and an additional therapeutic option to antiviral drugs since widespread drug resistance has been recently reported among influenza virus strains. Current FDA-approved anti-influenza viral drugs consist of the adamantane compounds (amantidine/rimantidine) and the neuraminidase inhibitors, oseltamivir and zanamivir [33], [34]. Widespread adamantine resistance was documented among seasonal H1N1 and H3N2 strains, and a majority of clade 1 and some clade 2 H5N1 isolates from Southeast Asia [35], [36], [37], [38]. Oseltamivir-resistant H5N1 and H1N1 isolates have also been reported [39], [40], [41]. Importantly, we show that the effectiveness of virus-specific IgY surpassed that of antiviral drug zanamivir in prophylaxis and treatment. For example, single pre-infection or multiple post-infection treatment with virus-specific IgY was sufficient to protect 100% of animals from lethal infection (Figs 1, 2, and 3), while a much higher number of combined pre- and post- infection treatments with a high dose of zanamivir (24 h before infection, 4 h before infection, 4 h after infection, and then twice daily for 5 days beginning 24 h after infection) is required to protect 90% of animals from lethal infection [42]. Daily treatment with virus-specific IgY for 4 days beginning as late as 24 h after infection did not result in protection, but increased the mean survival day (data not shown). A similar effect was seen during the course of more intense multiple treatment with zanamivir, which was initiated 24 h after infection and continued twice daily for 5 days [42].

IgY are relatively stable. We found no change in protective activity after at least 13 months' storage at 4°C, and lyophilization does not affect activity, making production of IgY practical. The use of IgY immunotherapy has many advantages since IgY does not activate the human complement system or human Fc-receptors, which all are well-known cell activators and mediators of inflammation [43]. For the preparation of IgY from egg yolks, we chose the water dilution method as the method is simple, efficient, and does not require use of any toxic compounds or additives. Such IgY preparations by this method have been used in other human studies [11], [31], and they contain low levels of egg cholesterols and triglycerides [18]. Another advantage is that other egg proteins found in IgY preparations could have additional positive antimicrobial and immuno-stimulatory effects [44]. Finally, as eggs are a component in the diet of many people, there is minimal risk of toxic side effects, except for those who are allergic to eggs.

Thus, we show that consumable eggs available in the markets of countries that impose mandatory H5N1 mass vaccination of poultry, offer an enormous source of valuable, affordable, and safe virus-specific IgY, which can be used for prevention and protection against potential H5N1 pandemic influenza. Our study provides a proof-of-concept for use of influenza virus-specific IgY in passive immunization against influenza outbreaks, including current H1N1 pandemic.
